# Analgesic Potential of Essential Oils

**DOI:** 10.3390/molecules21010020

**Published:** 2015-12-23

**Authors:** José Ferreira Sarmento-Neto, Lázaro Gomes do Nascimento, Cícero Francisco Bezerra Felipe, Damião Pergentino de Sousa

**Affiliations:** 1 Departamento de Ciências Farmacêuticas, Universidade Federal da Paraíba, CEP 58.051-900 João Pessoa-PB, Brazil; ferreira_system@hotmail.com (J.F.S.-N.); lazarofarm2@gmail.com (L.G.N.); 2 Departamento de Biologia Molecular, Universidade Federal da Paraíba, CEP 58.051-900 João Pessoa-PB, Brazil; cicero@dbm.ufpb.br

**Keywords:** essential oils, aromatic plants, natural products, analgesic, antinociceptive, pain, formalin, monoterpenes, phenylpropanoids, medicinal plants

## Abstract

Pain is an unpleasant sensation associated with a wide range of injuries and diseases, and affects approximately 20% of adults in the world. The discovery of new and more effective drugs that can relieve pain is an important research goal in both the pharmaceutical industry and academia. This review describes studies involving antinociceptive activity of essential oils from 31 plant species. Botanical aspects of aromatic plants, mechanisms of action in pain models and chemical composition profiles of the essential oils are discussed. The data obtained in these studies demonstrate the analgesic potential of this group of natural products for therapeutic purposes.

## 1. Introduction

Pain is an unpleasant sensation usually caused by intense or damaging stimuli. It is also defined as an unpleasant sensory or emotional experience associated with actual or potential tissue damage [1]. Pain is described as a multidimensional experience with many components involved and having motivational, emotional, sensory-discriminative, affective and cognitive aspects [2,3]. To assess pain and preclinically evaluate analgesic drugs, many irritating chemical agents can be used as nociceptive stimuli [3,4]. They induce a tonic pain state, which is evaluated by behavioral scoring. In the writhing test, the irritating agents are administered intraperitoneally, inducing a behavior stereotypical of abdominal contractions, which are quantified [3]. The formalin test in mice is a valid and reliable model of nociception, being sensitive for various classes of analgesic drugs. Hunskaar and Hole [5] described the test for use in mice. The response to formalin stimulus was studied during the first hour after formalin injection and an “early response” or a “late response” were described. A similar time course has also been observed in the original study in rats and two types of pain were postulated: a short-lasting pain caused by a direct effect on nociceptors, followed by a longer lasting pain due to inflammation [6]. Nociceptive tests may use chemical, electrical, mechanical, or thermal stimuli [3]. The hot-plate test is commonly used to investigate nociception and analgesia in rodents. The standard method as described by Woolfe and MacDonald [7] and modified by Eddy *et al.* [8], records latency for nociceptive responses in animals placed on a plate and kept at a constant temperature, usually about 55 °C. The analgesic effects of morphine and other narcotic analgesics are easily identified using this test. The tail flick is one of the oldest nociceptive tests [9]. The tail flick is a spinal reflex, but it is subject to supraspinal influences [10,11]. The test is highly sensitive to opiates [3]. Following tissue damage, as in autoimmune diseases, or with exposure to irritating agents, the immune system releases inflammatory mediators that activate and sensitize the nociceptive system [12]. Some inflammatory pain models rely on the administration of substances that induce an immune response (carrageenan, zymozan) [13], or on administration of these inflammatory mediators themselves [4].

Sometimes for the diagnosis of several diseases, pain is the only symptom. Throughout the history, man has used many forms of therapy for pain relief, among which, medicinal plants are highlighted due to their widespread and popular use. An example is *Papaver somniferum*, from which morphine was isolated. Though morphine is considered the prototype of opioid analgesics, it presents considerable side effects such as respiratory depression, sleepiness, decreased gastrointestinal motility, nausea and endocrine and autonomic nervous systems disorders [14]. The discovery of natural compounds that have similar analgesic activity, yet with fewer side effects is pertinent.

Plants producing essential oils belong to various genera distributed within 60 families. Selected families such as Alliaceae, Apiaceae, Asteraceae, Lamiaceae, Myrtaceae, Poaceae and Rutaceae are well known for their ability to produce essential oils of industrial and medicinal value [15,16]. Essential oils extracted from plants are highly concentrated mixtures of chemicals, both volatile and hydrophobic. The main chemical constituents present in essential oils are the monoterpenes, sesquiterpenes, and phenylpropanoids [14]. Many essential oils present diverse pharmacological properties, such as antimicrobial, anticonvulsant, hypnotic, anxiolytic, or anticancer [14,17,18]. Recent studies have highlighted the monoterpenes present in certain essential oils, such as menthol, linalool [19], limonene [20], myrcene [21] and 1,8-cineole [22]. Such essential oils have presented biological activities in differing animal models that include analgesic-like activity [23]. The objective of this work was to analyze studies involving the essential plant oils present in species with antinociceptive activity in animal models of nociception.

## 2. Methodology

The search was conducted in the scientific database PubMed, focusing on works published during the last six years (January 2009 to December 2014). The data were selected using the following terms: “essential oils” and “antinociceptive” or “analgesic” as well as the names of experimental models of nociception in animals such as “writhing”, “formalin”, “tail-flick”, “tail immersion” and “hot plate model”.

## 3. Results and Discussion

A large number of behavioral observation methods have been developed in order to study both nociception and the action of various analgesic drugs in animals [5,24]. In the present work, the antinociceptive activities of essential oils were evaluated in rats and/or mice (Table 1). A lack of clinical studies was observed due to the need for more extensive pre-clinical investigations on toxicity and safety of such essential oils. Acetic acid-induced writhing was performed in 72.2% of the studies, followed by formalin (66.7%), hot plate (27.8%), tail flick (11.1%) and tail immersion (5.6%) tests. The carrageenan test associated with inflammatory pain was present in 22.2% of the studies. According to the criteria used in the present work, we selected 31 plant species that produce essential oils and which were tested for antinociceptive activity (Table 1).

**Table 1:** Plants essential oils with analgesic-like activity.

Plant Specie	Major Constituent	Animal Model	Mechanism of Action	Reference
*Bunium persicum*	γ-Terpinene (46.1%)	Acetic acid-induced writhings, Formalin	Peripheral and central	[25]
*Citrus limon*	Limonene (52.77%)	Acetic acid-induced writhings, Formalin, Hot-plate	Central	[26]
*Cymbopogon citrates*	Myrcene (27.83%)	Acetic acid-induced writhings, Tail-flick	Not informed	[27]
*Cymbopogon winterianus*	Geraniol (40.06%)	Acetic acid-induced writhings, Formalin, Hot-plate	Peripheral and central	[28]
*Eucalyptus citriodora*	Citronellal (83.50%)	Acetic acid-induced writhings, Tail-flick	Not informed	[27]
*Eugenia caryophyllata*	Eugenol (87.34%)	Formalin, Tail-flick	Opioid	[29]
*Heracleum persicum*	Hexyl butyrate (56.5%)	Acetic acid-induced writhings, Formalin	Peripheral	[30]
*Hofmeisteria schaffneri*	Hofmeisterin III	Acetic acid-induced writhings, Hot-plate	Opioid	[31]
*Hyptis fruticosa*	1,8-Cineole (18.70% in leaves)	Acetic acid-induced writhings, Formalin	Peripheral and central	[32]
	α-Pinene (20.51% in flowers)			
*Hyptis pectinata*	β-Caryophyllene (40.90%)	Acetic acid-induced writhings, Formalin, Hot-plate	Peripheral and central (opioid, nitrergic and cholinergic)	[33]
*Illicum lanceolatum*	Myristicin (17.63%)	Acetic acid-induced writhings	Peripheral	[34]
*Lippia gracilis*	Thymol (24.08%)	Acetic acid-induced writhings	Peripheral	[35]
	Carvacrol (44.43%)	Acetic acid-induced writhings, Formalin, Hot-plate	Peripheral and central (opioid, nitrergic and cholinergic)	[36]
*Matricaria recutita*	α-Bisabolol oxide B (25.5%)	Carrageenan-induced mechanical hypernociception	Peripheral	[37]
*Mentha* x *villosa*	Piperitenone oxide	Acetic acid-induced writhings, Formalin, Hot-plate, Tail-flick	Peripheral	[38]
*Nepeta crispa*	Not informed	Formalin, Tail-flick	Not informed	[39]
*Ocimum basilicum*	Linalool (69.54%)	Acetic acid-induced writhings, Formalin, Hot-plate	Peripheral and Central (opioid)	[40]
*Ocimum gratissimum*	Eugenol (67.17%)	Formalin, Hot-plate	Central (opioid)	[41]
*Ocimum micranthum*	(*E*)-methyl cinnamate (33.6%)	Acetic acid-induced writhings, Formalin, Hot-plate	Peripheral	[42]
*Peperomia serpens*	(*E*)-Nerolidol (38.0%)	Acetic acid-induced writhings, Formalin, Hot-plate	Peripheral	[43]
*Pimenta pseudocaryophyllus*	Neral (27.59%) Geranial (36.49%)	Acetic acid-induced writhings	Peripheral	[44]
*Piper alyreanum*	Caryophyllene oxide (11.5%)	Formalin	Peripheral	[45]
*Satureja hortensis*	γ-Terpinen (50.5%)	Acetic acid-induced writhings, Formalin	Peripheral	[25]
*Senecio rufinervis*	Germacrene (40.19%)	Acetic acid-induced writhings, Hot-plate	Peripheral and central	[46]
*Tetradenia riparia*	14-hydroxy-9-epi-caryophyllene (18.27%–24.36%)	Acetic acid-induced writhings	Not informed	[47]
*Teucrium stocksianum*	δ-Cadinene (12.92%)	Acetic acid-induced writhings	Not informed	[48]
*Ugni myricoides*	α-Pinene (52.1%)	Carrageenan-induced mechanical hypernociception; CFA-induced mechanical hypernociception; Partial ligation of sciatic nerve	Not informed	[49]
*Valeriana wallichii*	δ-Guaiene (10%)	Acetic acid-induced writhings, Tail-flick	Peripheral	[50]
*Xylopia laevigata*	γ-Muurolene (17.78%)	Acetic acid-induced writhings, Formalin	Peripheral	[51]
*Vanillosmopsis arborea*	α-Bisabolol (70%)	Eye wiping (corneal nociception) Formalin	Peripheral and Central (TRVP1 cholinergic, adrenergic and serotoninergic)	[52]
*Zingiber oficinalle*	Zingiberene (31.08%)	Acetic acid-induced writhings	Peripheral	[53]
*Zingiber zerumbet*	Zerumbone (36.12%)	Acetic acid-induced writhings, Formalin, Hot-plate	Peripheral and central (opioid)	[54]
	Not informed	Acetic acid-induced writhings, Formalin	Peripheral and Central (TRVP1 glutamatergic, nitrergic and ATP-sensitive K^+^ channel blockade)	[55]

### 3.1. Bunium persicum Essential Oil

*Bunium persicum* (Boiss) B. Fedtsh is a grassy plant of the Apiaceae family with the common name of wild Caraway. The fruits contain about 2% (*w*/*v*) essential oil; caryophyllene, γ-terpinene and cuminyl acetate are the major components [56]. In the study by Hajhashemi [25], GC/MS analysis identified gamma-terpinene (46.1%) as the main component present in the essential oil of the fruits. The oil (at 100, 200 and 400 µL/Kg, p.o.) was tested in animal models of nociception and presented antinociceptive activity in the acetic acid-induced writhing and formalin (both phases) tests. According to Hajhashemi [25], the antinociceptive effect of *Bunium persicum* (Boiss) B. Fedtsh essential oil (fruit) depends on central mechanisms. Acetic acid acts indirectly by inducing the release of endogenous mediators, which stimulate nociceptive neurons sensitive to non-steroidal anti-inflammatory drugs (NSAIDS) and opioids [57]. Also, in the formalin test, the early phase seemed to be caused predominantly by C-fiber activation due to the peripheral stimulus, while the late phase appeared to be dependent on the combination of an inflammatory reaction in the peripheral tissue and functional changes in the ventral horn of the spinal cord [25]. Since essential oil of *B. persicum* was effective in suppression of the first phase of formalin test, it seems that at least a part of analgesic activity of *B. persicum* fruits is mediated centrally [25].

### 3.2. Citrus limon Essential Oil

The plants of the family Rutaceae (with approximately 2000 species) comprise 150 genera, the largest of which are *Citrus* (about 70 species) and Terminalia (about 200 species). *Citrus limon* (L.) Burm is a plant from north-northeastern Brazil and is known by the popular name of “limoeiro” [58,59]. In the study by Campêlo *et al.* [26], essential oil from the leaves of *Citrus limon* (50, 100 and 150 mg/Kg, p.o.) was tested using acetic acid-induced writhing, formalin and the hot plate test. GC-MS analysis revealed a mixture of monoterpenes, with limonene (at 52.77%) being the main component of the oil. Administration of this essential oil reduced the number of writhings, significantly inhibited the licking response to the formalin (only in the first phase of the test) and prolonged the delay in response time when mice were subjected to nociceptive stimulus in the hot plate test. It is important to note that the antinociceptive effect of the *Citrus limon* essential oil in the acetic acid-induced writhings and hot plate tests was partially reversed by naloxone (1.5 mg/Kg, i.p.), an opioid antagonist. Acetic acid-induced abdominal constriction is a standard, simple and sensitive test for measuring analgesia induced by both opioids and peripherally acting analgesics [24]. However, in the formalin test, the first phase is generated in the periphery through the activation of nociceptive neurons by direct action of formalin. The second phase occurs through the activation of the ventral horn neurons at the spinal cord level. Morphine, a typical narcotic drug, inhibits nociception in both phases, but drugs with peripheral activity such as indomethacin and corticosteroids, inhibit only the second phase [60].

### 3.3. Cymbopogon citrates and Cymbopogon winterianus Essential Oils

The *Cymbopogon* genus (Poaceae), found in tropical countries, is composed of more than 100 species [61]. About 56 of these are aromatic and some have medicinal, pharmacological and industrial importance [62]. Two species of *Cymbopogon*: *Cymbopogon nardus* (Jamarosa) and *C. winterianus* (Java citronella) are known to have similar volatile oil scents and medicinal uses, but they reveal different contents. GC-MS analysis has shown that myrcene (27.83%), geranial (27.0%) and neral (19.9%) were the major components present in the essential oil of the leaves of *Cymbopogon citrates* [27]. This essential oil (2000 and 3000 mg/Kg, p.o.) was tested in the acetic acid-induced writhings and tail flick models of nociception. Despite the plant material presenting antinociceptive effect, the authors did not suggest any mechanism for the action of the oil. HPLC and GC-MS analyses developed [28] indicated the presence of geraniol (40.06%) as the major component present in the essential oil of the leaves of *Cymbopogon winterianus*. Gbenou *et al.* [27] tested the plant material (at 50, 100 and 200 mg/Kg, p.o.) in three different animal models of nociception (acetic acid-induced writhings, hot plate and formalin tests). In the acetic acid-induced writhing and formalin tests, the essential oil significantly reduced the number of writhings and paw licking times (in both phases). In contrast, the material did not alter the latency time for mice licking of the rear paws in the hot-plate test. It is known that acetic acid-induced abdominal writhing causes algesia through liberation of endogenous substances, which excites pain nerve endings [63]. Increased levels of PGE_2_ and PGF_2α_ in the peritoneal fluid have been reported as responsible for the pain sensations caused by intraperitoneal administration of acetic acid [64]. Based on these results, the authors assume that the mode of action of the essential oil might involve, at least in part, a peripheral mechanism. In addition, the formalin model of nociception discriminates pain in its central and peripheral components [65]. The test consists of two different phases separated in time: the first one is generated in the periphery through activation of nociceptive neurons through direct formalin action and the second phase occurs through the activation of ventral horn neurons at the spinal cord level. Morphine, a typical narcotic drug, inhibits nociception in both phases [60], but drugs with peripheral action, such as indomethacin and corticosteroids, inhibit only the second phase. Moreover, drugs such as acetylsalicylic acid and paracetamol, which inhibit prostaglandin synthesis, block only the second phase of the formalin test [6,24]. Finally, the authors conclude that mild analgesics (such as aspirin) lack antinociceptive action in thermal tests such as the hot-plate test, but have significant antinociceptive activity in tonic tests (writhing and formalin tests), which are characterized by direct chemical stimulation of nociceptors. Since it has been reported that thermal and tonic tests elicit selective stimulation of A-γ fibers and C fibers, respectively [66], essential oil from the leaves of *Cymbopogon winterianus* may interfere with the transmission of both fibers, or a single common pathway.

### 3.4. Eucalyptus citriodora Essential Oil

Eucalyptus citriodora is an aromatic medicinal plant species belonging to the family of Myrtaceae. The antinociceptive effect of the essential oil extracted from the leaves of *Eucalyptus citriodora* was assessed [27]. GC-MS analysis indicates that citronellal (83.50%) is the chief constituent of the essential oil. The essential oil (at 1200 and 1800 mg/Kg, p.o.) was tested in the acetic acid-induced writhings and tail-flick models of nociception. Despite the fact that in both tests the plant material presented antinociceptive effect, the authors did not suggest any mechanism for the antinociceptive action of the oil.

### 3.5. Eugenia caryophyllata Essential Oil

*Eugenia caryophyllata* belongs to the Myrtaceae family [67]. The major component of *Eugenia caryophyllata* essential oil is eugenol with lower amounts of betacaryophyllene and eugenyl acetate [68,69]. In work developed by Halder *et al.* [29], GC-MS analysis confirmed that eugenol (87.34%) is the main compound present in the essential oil extracted from the dried flower buds of *Eugenia caryophyllata.* In this work, Halder *et al.* [29] tested the oil (0.025, 0.050 and 0.1 mL/Kg, i.p.) in formalin and tail-flick tests. In the formalin test, *Eugenia caryophyllata* essential oil (0.1 mL/Kg) was effective in reducing the duration of licks in the first phase. However, in the second phase, the pain response was reduced at all the doses. The plant material was observed to show variable effects in modifying pain response in the tail-flick experiment. At the 0.1 mL/Kg dose, the material significantly decreased the tail-flick latency thus showing hyperalgesic effect. On the contrary, the dose of 0.025 mL/kg increased the mean tail-flick latency compared to control group. It is important to emphasize that this effect was reversed by naloxone (1 mg/Kg, i.p.). It is well established that the tail-flick experiment evaluates the central mechanisms of pain relief, as observed in the case of opioids like morphine [70]. It has been demonstrated in previous studies that eugenol (the chief constituent of the oil) probably acts by both opioid receptors and alpha adrenergic receptors [71]. Morphine which acts on the opioidergic receptors, at low dosages (subanalgesic) produces an enhanced sensitivity to the noxious stimulus (hyperalgesia) in the tail-flick test [72]. It is possible that *Eugenia caryophyllata* essential oil is active on the opioid receptors. However, the variable response seen at different doses could also be due to the effect of other constituents present in the oil apart from eugenol [29].

### 3.6. Heracleum persicum Essential Oil

The genus *Heracleum* belongs to the Apiaceae (Umbelliferae) family and includes more than 70 species from all around the world [73]. For *Heracleum persicum* the essential oil is one of the most important constituents for the fruits [74], leaves [75], flowers [76] and roots [77]. According to the GC/MS analysis carried out by [30], hexyl butyrate (56.5%) is the major constituent of the essential oil of the fruits of *Heracleum persicum.* In this study, Hajhashemi *et al.* [30] tested the essential oil (50 and 100 mg/Kg, p.o.) in the formalin and acetic acid-induced writhing tests. The antinociceptive effect of the essential oil was observed in both tests. However in the formalin test, the material was devoid of effect in the first phase of the model. In the acetic acid test, (although the pain in the abdominal writhes is not model specific), the involuntary muscle twitches of the abdomen may be of interest because of their similarity those known in visceral disorders [3,78]. In the formalin pain model, oral administration of the oil reduced the late phase behavioral response to s.c. formalin injection in mice and since the late phase is inflammatory in origin, it indicates the peripheral antinociceptive effect of the plant material [30].

### 3.7. Hofmeisteria schaffneri Essential Oil

*Hofmeisteria schaffneri* (A. Gray) R.M.King & H.Robinson belongs to the Asteraceae family [31]. Previous chemical work with the plant allowed isolation of several thymol and northymol derivatives [79,80], including Hofmeisterin III, thymol itself and 8,9-epoxy-10-acetoxythymyl angelate. Angeles-López *et al.* [31] tested the essential oil (1–100 mg/Kg, p.o.) obtained from the dried aerial parts of *Hofmeisteria schaffneri*, along with the major component [thymol (10–100 mg/Kg, p.o.)], using the hot plate test. Two other thymol derived compounds were synthesized and tested as well: thymyl isovalerate (0.1–17.7 mg/Kg, p.o.) and thymyl isobutyrate (0.1–17.7 mg/Kg, p.o.). Hofmeisterin III significantly increased latency to thermal stimuli. The antinociceptive effect of the compound was not reversed by pre-treatments with either glibenclamide (10 mg/Kg, i.p.), a K^+^ channel blocking, or l-NAME (30 mg/Kg, i.p.), an inhibitor of nitric oxide synthase. However, pre-treatment with naloxone (1 mg/Kg, i.p.), a non-selective opiate receptor antagonist partially reversed the effect of Hofmeisterin III in the hot plate test. All of the other compounds showed similar effects, except 8,9-epoxy-10-acetoxythymyl angelate. The authors suggest that esterification of thymol’s free phenolic group may reduce the activity of the compound. However, thymol was only active at the highest dose. It has been previously shown that thymol partially blocks voltage-operated Na^+^ [81] and K^+^ [82] channels and directly activates aminobutyric acid GABA_A_ receptors [83]. Thymol also reversibly inhibited prostaglandin synthesis; probably related to the analgesic effect of thymol in endodontic therapy [84]. Furthermore, it was speculated that thymol could have an analgesic effect due to its agonistic effect on adrenergic receptors [85].

### 3.8. Hyptis fruticosa and Hyptis pectinata (L.) Poit Essential Oils

The genus *Hyptis* (Lamiaceae) consists of approximately 400 species distributed from the southern United States to Argentina and exhibits a major morphological diversity in the Brazilian Cerrado [86]. In Brazil, such plants are frequently found on the northeastern coast [87]. Franco *et al.* [32] studied the antinociceptive effect of the essential oil (25, 50 and 100 mg/Kg, i.p.) extracted from *Hyptis fruticosa* leaves and flowers in the acetic acid-induced writhing and formalin tests. Both oil samples presented the same major constituents, however, in different proportions: 1,8-cineole (18.70% and 12.46%, respectively), α-pinene (12.29% and 20.51%, respectively) and β-pinene (8.56% and 13.54%, respectively). In a dose-dependent manner, both oils reduced the number of acetic acid i.p. administration induced writhing movements. The effects were not reversed by naloxone. In addition, the essential oils significantly reduced the licking time in the first and second phases of the formalin test. The acetic acid induced abdominal contraction test reveals peripheral activity, while the formalin method reveals both central and peripheral activities [88]. Drugs that act primarily on the central nervous system inhibit both phases of the formalin test, while peripherally acting drugs inhibit the late phase [3]. The neurogenic phase (early phase) is probably a direct result of stimulation in the paw and reflects centrally mediated pain with the release of substance P, while the inflammatory phase (late phase) is due to the release of histamine, serotonin, bradykinin and prostaglandins [88]. In addition, studies performed with 1,8-cineole and β-pinene showed the centralized antinociceptive properties of these monoterpenes on hot plate and tail-flick tests. It was also demonstrated that β-pinene may well be considered a partial agonist of opioid μ receptors, while 1,8-cineole seems not to participate in this family of receptors [89]. Franco *et al.* [32] suggested, therefore, that the essential oils have both peripheral and central analgesic actions without opioid system influence, although the central activity was more discrete. In a study, the analgesic effect of the essential oil (10, 30 and 100 mg/Kg, p.o.) obtained from the leaves of *Hyptis pectinata* was tested using acetic acid-induced writhing, formalin, and hot plate tests [33]. GC-MS analysis showed that β-caryophyllene (40.90%) and caryophyllene oxides (30.05%) were the main compounds present in the oil. In pharmacological tests, we observed that treatment with increased doses of *Hyptis pectinata* essential oil resulted in similar degrees of inhibition in contortions. Naloxone (1 mg/Kg, i.p.), an opioid antagonist, did not reverse the anti-hyperalgesic effect of the *Hyptis pectinata* essential oil at 30 mg/kg; however, l-NAME (3 mg/Kg, i.p.), an inhibitor of the NO system, and atropine (1 mg/Kg, i.p.), a cholinergic antagonist, showed significant effects in reducing the antinociceptive activity of the *Hyptis pectinata* essential oil using the acetic acid-induced writhing model. The essential oil also presented antinociceptive effect in the hot-plate model. In this test, naloxone, l-NAME, and atropine antagonists were able to inhibit the anti-hyperalgesic effect of the the *Hyptis pectinata* essential oil. When these same antagonists were screened for their ability to reverse the analgesic effect of essential oil in the formalin model, only atropine was able to maintain its effect. Naloxone and l-NAME did not reverse the antinociceptive effect of *Hyptis pectinata* essential oil. The mechanism of action of *Hyptis pectinata* essential oil was investigated by pre-treating animals with several drugs which interfere in different systems. The results demonstrate the involvement of the l-arginine-nitric oxide pathway in the antinociceptive effect of the essential oil, which is in accordance with others who have shown the system’s participation in antinociceptive effects during peripheral inflammation [90]. The involvement of the opioid system in the antinociceptive activity of *Hyptis pectinata* essential oil was evaluated by pre-treating mice with an opioid antagonist, naloxone. The results suggest that the anti-hyperalgesic effect observed in the hot plate model is due, in part, to the involvement of opioid system because naloxone reversed the antinociceptive activity of the essential oil.

### 3.9. Illicum lanceolatum Essential Oil

*Illicium lanceolatum* A.C. Smith (Illiciaceae family) is a popular Chinese aromatic plant. The leaves and roots of *Illicium lanceolatum* have often been used as traditional Chinese medicines to treat bruises, internal injuries and back pain. GC/MS analysis carried out [34] indicated the presence of the following phenylpropenes in the essential oil of the roots of *Illicium lanceolatum*: myristicin (17.63%), α-asarone (17.23%), methyl isoeugenol (11.19%), apiol (8.82%), isolongifolol (5.94%) and τ-cadinol (4.32%). The antinociceptive action of the essential oil was tested in animals subjected to the acetic acid-induced writhing test. According to Liang *et al.* [34], the essential oil produced inhibition of the writhing responses in inhibitory ratios of 26.60%, 31.73% and 35.90%, respectively. The results indicated that the essential oil from the roots of *Illicium lanceolatum* possesses significant analgesic activity. However, more investigations are needed in order to elucidate its mechanism of action.

### 3.10. Lippia gracilis Essential Oil

The genus *Lippia* (Verbenaceae) is widely distributed in tropical and subtropical America and Africa and consists of approximately 250 species of herbs, shrubs and small trees [91,92]. In Brazil, the genus *Lippia* is represented by nearly 120 species, conspicuous for their flash appearance during the blooming period and by their fragrance, in general, strong and pleasant [93]. *Lippia gracilis* Schauer (Verbenaceae), known in Brazil by the name “alecrim-da-chapada”, is an herb commonly found in Northeastern Brazil, it is highlighted because it presents high monoterpene contents [94], such as carvacrol, *o*-cymene, γ-terpinene and β-caryophyllene [95]. Several communities in northeastern Brazil use *Lippia gracilis* to treat cough, bronchitis, nasal congestion and headache [96]. It was investigated the analgesic effect of the essential oil from *Lippia gracilis* leaves obtained under water stress condition (50–200 mg/Kg) in mice subjected to the acetic acid writhing test [35]. A chemical analysis performed by the group indicated as the main constituents the presence of thymol (32.68%), *p*-cymene (17.82%), methyl thymol (10.83%), carvacrol (7.53%), γ-terpinene (7.13%), β-caryophyllene (6.47%), 1,8-cineole (3.45%), and myrcene (3.35%). Oral administration of the essential oil caused inhibition of acetic acid-induced writhes at the doses of 50, 100 and 200 mg/Kg. Mendes *et al.* [35] affirm that essential oil from the leaves of *Lippia gracilis* displays antinociceptive action, possibly by inhibiting the release of endogenous mediators that stimulate the nociceptive neurons [57]. Further studies accomplished by [36] confirmed the antinociceptive effect of *Lippia gracilis* (leaf) essential oil, as previously described by Mendes and collaborators. In this work, the essential oil (10, 30 and 100 mg/kg, p.o.) was tested in mice subjected to the acetic acid-induced contortion, formalin-induced licking and hot plate tests. The chemical analysis indicated the presence of carvacrol (44.43%), *o*-cymene (9.42%), γ-terpinene (9.16%) and β-caryophyllene (8.83%) as the major constituents of the essential oil. In the acetic acid-induced contortion test, the mice treated with increasing doses of *Lippia gracilis* essential oil showed inhibition of contortions with doses of 10, 30, or 100 mg/kg. The ability to reduce acetic acid-induced writhings and formalin-induced licking responses are indicative of antiinflammatory effect. The acetic acid-induced writhings model has been used as a screening tool for the assessment of analgesic or antiinflammatory agents [57]. Guilhon *et al.* [36] postulated that acetic acid acts by inducing the release of mediators that stimulate nociceptive neurons sensitive to non-steroidal antiinflammatory drugs and narcotics. The mediators (*i.e*., histamine, serotonin, bradykinin and others) released into the peritoneal fluid cause an increase in vascular permeability, reducing the threshold of nociception and stimulating nociceptive fibres’ nervous terminals [64,97,98]. To confirm the peripheral anti-hyperalgesic effect, Guilhon *et al.* [36] used the formalin model. In this model, at the doses tested (10, 30, or 100 mg/Kg) *Lippia gracilis* essential oil did not reduce the time that the animal spent licking the formalin-injected paw (first phase). All doses of the essential oil however significantly reduced the licking time in the second phase after the formalin injection. Centrally acting drugs such as narcotics inhibit both phases of the nociceptive response equally [60]. Drugs with peripheral action, such as aspirin and dexamethasone inhibit only the second phase [6,24]. The antinociceptive effect of the essential oil was also tested in the hot plate model, where pre-treatment with the oil (10–100 mg/kg) resulted in significant anti-hyperalgesic activity (all doses tested only in the late phase). In order to evaluate a possible mechanism underlying the antinociceptive effect of *Lippia gracilis*, it was assessed the involvement of the opioid, cholinergic and nitric oxide (NO) systems in the essential oil effects observed following administration. The opioid antagonist naloxone (1 mg/Kg, i.p.) did not reverse the anti-hyperalgesic effect of *Lippia gracilis* (at 30 mg/Kg), for either acetic acid-induced writhings or formalin induced licking. Atropine (1 mg/Kg, i.p.), the cholinergic antagonist significantly reduced the antinociceptive activity of the essential oil in both models. Similarly, the nitric oxide synthase inhibitor, l-NAME (3 mg/Kg, i.p.), slightly reduced the *Lippia gracilis* induced anti-hyperalgesia. The three antagonists were able to inhibit the anti-hyperalgesic effect of *Lippia gracilis* in the hot plate model. These results suggest that constituents from essential oil may be acting through different pathways to produce the observed antinociceptive activity. As such, it is likely that the mechanisms underlying this activity are multi-fold and require more investigation [36].

### 3.11. Matricaria recutita L. Essential Oil

*Matricaria recutita* L. is an herbaceous, annual, aromatic plant, native in Southern and Eastern Europe [99]. Matricaria is widely distributed and cultivated has been used in traditional medicine since the time of ancient Egypt, Greece and Rome [100]. The matricaria flower is a well-known remedy for various gastrointestinal problems: spasms, inflammatory diseases, disorders such as indigestion, flatulence, excess gas production and bloating. Matricaria preparations are used externally in medicine and cosmetics, owing to its antiinflammatory properties [99]. Most of the pharmacological properties of the matricaria flower are related to its essential oil containing (α-bisabolol and its oxides; chamazulene and spiroether) and flavonoids such as apigenin and apigenin 7-*O*-glucoside and sesquiterpene lactones such as matricin and coumarins [99,101]. Tomić *et al.* [37] investigated the antinociceptive effect of bisabolol-oxides of matricaria oil (25, 50 and 100 mg/kg, p.o.) in mice subjected to the carrageenan-induced inflammation test, which was measured in a modified “paw-pressure” test as previously described by [102]. GC/MS analysis indicated the presence of α-bisabolol oxide A (21.5%), α-bisabolol oxide B (25.5%) and (*Z*)-spiroether (*cis*-en-yn-spiroether) (10.3%) as the main components. Matricaria oil produced a significant dose-dependent reduction of hyperalgesia induced by carrageenan in both prophylactic and therapeutic treatment schemes. In post carrageenan injection, it is known that various inflammatory mediators are involved in nociception and hyperalgesia: histamine, 5-hydroxytryptamine (5-HT), prostaglandins (PGs) and others [103]. In this work, Tomić *et al.* [37] suggest that the antihyperalgesic effect of matricaria oil is most likely related to its two main components-α-bisabolol oxides A and B. This affirmation is based on Rocha [104] who demonstrated in mice that (−)-α-bisabolol (p.o.) diminished mechanical hyperalgesia of the carrageenan paw injected and reduced nociceptive behavior in a second, inflammatory phase of the formalin test. However, [105] demonstrated *in vitro* that α-bisabolol oxides A and B do not inhibit cyclooxygenase (COX), an enzyme responsible for prostaglandin synthesis. It was shown that *cis*-en-yn-spiroether and (−)-α-bisabolol both act as COX inhibitors [106] and that (−)-α-bisabolol reduces neuronal excitability in mice sciatic nerves, probably by an irreversible blockade of voltage-dependent sodium channels [107]. Tomić *et al.* [37] conclude, therefore, that bisabolol oxides could decrease neuronal excitability similarly to (−)-α-bisabolol; and seeing that *cis*-en-yn-spiroether and (−)-α-bisabolol can inhibit COX, the reduction of hyperalgesia exerted by matricaria oil may be explained by the reduced neuronal excitability and, in part, by inhibition of prostaglandin synthesis.

### 3.12. Mentha x villosa Huds Essential Oil

Various mentha species are used all over the world as choleretic, spasmolytic and analgesic agents [108]. In Northeastern Brazil, *Mentha* x *villosa* Huds (Labiatae), an aromatic herb, is widely used in folk medicine as a stomachic medicine, an anxiolytic agent, for the treatment of menstrual cramps and for diarrhea with cholic and blood in the stools [109]. Sousa *et al.* [38] investigated the antinociceptive effect of *Mentha* x *villosa* Huds leaves essential oil (at 10, 100 and 200 mg/Kg, p.o.) and its major constituent piperitenone oxide (also at 10, 100 and 200 mg/Kg, p.o.) and as determined by GC-MS analysis. Both substances were tested in mice subjected to the acetic acid-induced writhing, formalin, hot plate and tail-flick tests. In the first test, both substances reduced the number of writhings. At the lower doses (10 and 100 mg/kg body weight), neither agent induced significant changes in the number of writhings. The association with naloxone (2 mg/Kg, s.c.) did not alter the number of writhings as compared to either group treated with essential oil or piperitenone oxide alone. The antinociceptive effect of *Mentha* x *villosa* Huds leaves essential oil (and its major constituent) was assessed in the formalin test. In this model, both agents reduced significantly the paw licking time for the second phase of the formalin test only. The effect was not reversed by naloxone (2 mg/kg, s.c.). At dosages lower than 50 mg/kg body weight, neither agent induced significant changes in the second phase of testing. In the hot plate and tail flick tests, neither agent presented antinociceptive effect. The hot-plate and tail immersion tests are reported to be useful tests for discriminating analgesic agents acting at the spinal medulla level (primarily) and at the higher central nervous system levels, from those acting by peripheral mechanisms, with positive results indicating central activity [3]. Sousa *et al.* [38] affirm that according to the acetic acid-induced writhing, both the essential oil from *Mentha* x *villosa* Huds leaves and piperitenone oxide act by peripheral mechanisms. Also, based on the results of the hot-plate and tail immersion tests, the authors assumed that the antinociceptive effects of both agents were not related to central processing.

### 3.13. Nepeta crispa Willd. Essential Oil

*Nepeta crispa* Willd. is a plant of the Lamiaceae family and one of the aromatic and medicinal plants of Iran [110]. In Iranian folk medicine, especially in the Hamadan province, distillates and infusions are prepared from its aerial parts and are traditionally used as a sedative, relaxant and carminative and also as a restorative tonic for nervous and respiratory disorders [111]. Ali *et al.* [39] evaluated the antinociceptive activity of the essential oil of *Nepeta crispa* (30, 100 and 200 mg/Kg, i.p.) in rats subjected to the tail-flick and formalin test pain models. The administration of the essential oil of *Nepeta crispa* presented antinociceptive effect in the tail-flick and formalin (in both phases) tests. Such effect, however, needs more investigation in order to elucidate the possible mechanism of the antinociceptive action of the essential oil.

### 3.14. Ocimum basilicum, Ocimum gratissimum and Ocimum micranthum Essential Oils

*Ocimum* (Lamiaceae) is a genus that comprises more than 150 species; these are distributed in tropical and subtropical regions [112,113,114]. The essential oil of many Ocimum species is used to treat headaches, diarrhea, helminth infestations, inflammations and pain [115,116,117]. It is also used in the pharmacy, perfumery and cosmetics industries because of its odorant, bactericide, fungicide and insect repellent properties. Chromatographic essential oil analyses have shown that plants from this genus are rich in volatile constituents such as linalool, geraniol, citral, alcanfor, eugenol, thymol, 1,8-cineole and neryl acetate [112,116]. The antinociceptive effect of the essential oil from the leaves of *Ocimum basilicum* was evaluated [40]. In this work [40], the essential oil (50, 100 and 200 mg/Kg, s.c.) in mice subjected to acetic acid-induced writhing, hot plate and formalin tests was tested. GC-MS analysis indicated the presence of the following major constituents: linalool (69.54%) and geraniol (12.55%). In the acetic acid-induced writhing test, the essential oil reduced the writhings in a dose-dependent manner. The intraperitoneal administration of acetic acid irritates the gastric serous membrane and produces abdominal writhings due to inflammation, which is the peripheral component of pain. The action of anti-inflammatory substances such as indomethacin results in inhibition of the enzyme cyclooxygenase in the arachidonic acid pathway, preventing the biosynthesis of prostaglandins and prostacyclins and reducing pain [3,118]. In the hot plate test, the essential oil at 50 mg/kg significantly increased the mice response times to thermal stimulus. This effect was reversed in the presence of naloxone (5 mg/Kg, i.p.), which suggests that opioid receptors are involved in the essential oil antinociceptive action [118,119,120]. Finally, the essential oil’s effects on the first and second phases of pain during the formalin test could be characterized by the reduction of the paw licking time after stimulus. The results suggested that action on the central and peripheral components of pain might be involved. It is known that the first phase of pain is neurogenic and occurs through nociceptive neuronal activity by direct action. The second phase occurs through ventral horn neuronal activity at the spinal cord level, which is characterized by inflammation and sensitivity to NSAIDs [118,120]. Drugs that present central action, such as narcotics (morphine), inhibit both phases of pain, while peripheral drugs only inhibit the second phase [60]. Venâncio *et al.* [40] suggest that the antinociceptive activity of the essential oil of *Ocimum basilicum* seems to be associated with linalool (the main constituent), acting on K^+^-ATP channels, which has an important role in pain modulation [121]. In a later work, the antinociceptive effect of *Ocimum gratissimum* essential oil (30, 100 and 300 mg/Kg, p.o.) and its major components (at 5 and 10 mg/Kg, p.o.) were evaluated [41]. Phytochemical analysis confirmed the presence of eugenol (67.17%) and myrcene (0.24%) as the main constituents of the oil. Initially, mice were subjected to the formalin pain model. In this test, animals treated with the essential oils eugenol and myrcene showed reduced licking times of the paw in the first (5–10 min) and second (15–30 min) phases of nociception. On the other hand, in the hot-plate test, the essential oil increased the latency of paw withdrawal from the hot plate, even after 4 h of administration. Animals that received 5 or 10 mg/Kg of eugenol or 5 or 10 mg/kg of myrcene exhibited a significant increased latency to either lick the paw(s), or to jump from the hot plate. Pre-treatment with naloxone (1 mg/Kg, i.p.) significantly reversed the antinociceptive effects of the oil, eugenol and myrcene in the hot plate test. These data suggest that the opioid system is involved in the mediation of the antinociceptive effects of *Ocimum gratissimum* essential oil and its isolated active principles, eugenol and myrcene. Opioid receptors (m, к and d) are located in several steps of the pain transmission pathway and are responsible for the direct and indirect antinociceptive activities of opioid agonists [122]. Substances that can also activate these receptors should be of great pharmacological and therapeutic importance [123].Antinociceptive effects of *Ocimum micranthum* essential oil (15, 25, 50 and 100 mg/Kg, p.o.) were studied by Pinho *et al.* [42] in mice subjected to the following pain models: acid-induced writhing, formalin and hot-plate tests. Chemical analyses of the essential oil revealed the following composition: (*E*)-methyl cinnamate (33.6%), limonene (12.9%), carvone (9.6%), β-caryophyllene (8.03%), linalool (7.2%), (*Z*)-methyl cinnamate (5.92%), β-selinene (3.95%), α-selinene (2.82%), α-humulene (2.7%) and *trans*-α-bergamotene (2.68%). Initially, *Ocimum micranthum* essential oil significantly reduced the acetic acid-induced writhing responses. Under formalin-induced nociception, only the second phase was significantly inhibited. No effect was observed in mice subjected to the hot-plate test. Based on the lack of significant results in the first phase of the formalin test and in the hot plate model Pinho *et al.* [42] conclude that the essential oil inhibits nociception of inflammatory origin acting at the peripheral rather than supraspinal and/or spinal level. However, as β-caryophyllene is found in the composition of the essential oil, early studies showing that β-caryophyllene has both antinociceptive and anti-inflammatory actions [124,125,126] may be due to its cannabinoid receptor 2 (CB2) activating properties [127]. The formalin model of nociception may not be a reliable model to reveal the actions of substances outside of certain pharmacological profiles.

### 3.15. Peperomia serpens (Sw.) Loud Essential Oil

The genus *Peperomia*, belonging to Piperaceae, comprises an estimated 1500–1700 species [128]. *Peperomia serpens* (Sw.) Loud. In the Amazon rainforest it is known as “carrapatinho” or “carapitinha” growing wild on differing host trees. The decoction of its leaves is recommended for anti-inflammatory and analgesic properties, particularly against flu, asthma, cough, earache and irritation provoked by ant bites [129]. Other oils of *Peperomia* contain mono- and sesquiterpenes, as in the case of *Peperomia serpens*, whose main constituents were α-humulene, (*E*)-caryophyllene, (*E*)-nerolidol and (*Z*)-nerolidol acetate [130]. Pinheiro *et al.* [43] evaluated the antinociceptive effect of the essential oil from the whole plant (31.25, 62.5, 125, 250 and 500 mg/Kg, p.o.) in mice subjected to the acetic acid-induced writhing, formalin and hot plate tests. GC-MS analysis indicated the presence of the following major constituents: (*E*)-Nerolidol (38.0%), ledol (27.1%), α-humulene (11.5%), (*E*)-caryophyllene (4.0%) and α-eudesmol (2.7%). Oral pretreatment with *Peperomia serpens* (Sw.) Loud essential oil evoked dose-dependent inhibition of acetic acid-induced abdominal writhes in mice. The writhing test induced by acetic acid in mice is described as a typical model of study of inflammatory pain, used to screen when evaluating analgesics or anti-inflammatory drugs [131]. The local irritation provoked by intraperitoneal injection of acetic acid triggers the liberation a variety of mediators such bradykinin, substance P and prostaglandins, and especially PGI2, as well as some cytokines such as IL-1β, TNF-α and IL-8 [132]. Such mediators activate chemosensitive nociceptors that contribute to the development of this type of inflammatory pain, which is known to be sensitive to non-steroidal anti-inflammatory drugs (NSAIDs). Like indomethacin (10 mg/Kg, p.o.), the essential oil was able to reduce dose-dependent acetic acid-induced writhing response, suggesting a mechanism resulting in peripheral antinociceptive effect. Pretreatment of animals with the essential oil showed no antinociceptive effect in mice subjected to the hot-plate test. Such results indicate non-participation in thermal stimulation associated with central neurotransmission where heat activates nociceptors (Aδ, and C fibers), driving the momentum to the dorsal horn of the spinal cord subsequently to cortical centers. Finally, in the formalin test, the oil inhibited both first (neurogenic pain), and second (inflammatory pain) phases of the test. Such effect was not altered by naloxone pretreatment [43]. The formalin test is a very useful method for not only assessing antinociceptive drugs but also for helping to elucidate their action mechanism. Centrally acting drugs such as narcotics inhibited both phases equally. Peripheral acting drugs such as NSAIDs and corticoids inhibited mainly the second phase [60]. According to Pinheiro *et al.* [43], *Peperomia serpens* (Sw.) Loud essential oil induces its antinociceptive action by direct action on nociceptive afferent fibers not interacting with the opioid system. In addition, the oil was effective in the second phase of the formalin test indicating anti-inflammatory activity. These results suggest that the antinociceptive action of *Peperomia serpens* (Sw.) Loud essential oil is more related to a peripheral mechanism than a central one.

### 3.16. Pimenta pseudocaryophyllus (Gomes) L.R. Landrum Essential Oil

*Pimenta pseudocaryophyllus* (Gomes) L.R. Landrum (Myrtaceae) is a plant popularly known in Brazil as *pau-cravo, louro-cravo, louro, craveiro, craveiro-do-mato*, *chá-de-bugre and cataia* [133,134,135]. In folk medicine, the leaves have been used to produce a refreshing drink with calming, diuretic and aphrodisiac properties, as well as to treat colds with their complications, and digestive and menstrual problems [133,134,135]. De Paula *et al.* [44] studied the antinociceptive effect of the essential oil obtained from leaves of *Pimenta pseudocaryophyllus* in mice subjected to the acetic acid-induced writhing test. GC-MS analysis indicated the presence of oxygenated mono- and sesquiterpenes (69.65% and 13.7%, respectively), and monoterpene aldehydes neral and geranial were the major components (27.59% and 36.49%, respectively), which are referred to as citral when their isomers are mixed [44]. The essential oil at doses of 60, 200 and 600 mg/Kg, p.o., showed significant dose-dependent inhibitory effects on abdominal contortions induced by intraperitoneal acetic acid in mice. De Paula *et al.* [44] suggest that inhibition by *Pimenta pseudocaryophyllus* essential oil of the contortions induced by chemical stimulation in mice in this study may be due to both peripheral and central mechanisms. However, more investigations are needed to reinforce their case.

### 3.17. Piper alyreanum C.DC Essential Oil

*Piper alyreanum* C.DC, a member of the Piperaceae family, is a small tree that is widely distributed in tropical and subtropical regions, and greatly in North and South America. In Brazil, it is found in the North, mainly in the Amazon forest and is popularly known as “João brandinho”, “pimenta longa”, “pimenta longa da mata”, “pimenta de cobra” and “pani-nixpu”. Moreover, this plant has been used as an immunomodulator, analgesic and antidepressant in folk medicine [45]. The antinociceptive effect of the essential oil obtained from the aerial (leaves and stems) of *Piper aleyreanum* was evaluated. The essential oil (30, 100, 300 and 1000 mg/Kg, p.o.) was tested in mice subjected to the formalin test. GC-MS analysis indicated the presence of caryophyllene oxide (11.5%), β-pinene (9%), spathulenol (6.7%), camphene (5.2%), β-elemene (4.7%), myrtenal (4.2%), verbenone (3.3%) and pinocarvone (3.1%) as the major constituents [45]. The results indicated that the essential oil significantly inhibited both the neurogenic and inflammatory phases of formalin-induced licking. However, its antinociceptive effects were significantly more pronounced in the second phase of this pain model. Also, it was noted that pre-treatment with the non-selective opioid receptor antagonist naloxone (1 and 5 mg/Kg, i.p.) did not reverse the antinociception caused by the essential oil [45]. Nociception as produced by formalin (first phase) is quite resistant to the majority of NSAIDs, such as acetylsalicylic acid, indomethacin, paracetamol and diclofenac. However, these drugs can dose-dependently attenuate the second phase of formalin-induced licking [25,136,137]. Moreover, it has also been reported that morphine, some tachykinin receptor antagonists, non-selective excitatory amino acid antagonists and both B1 and B2 bradykinin receptor antagonists are able to inhibit both phases of the formalin test [138,139]. Lima *et al.* [45] suggest that the opioid system is unlikely to be involved in the antinociceptive action of *Piper alyreanum* essential oil. This is inferred by the fact that pre-treatment of animals with naloxone, a nonselective opioid receptor antagonist, did not inhibit the antinociceptive effect of morphine in the formalin model.

### 3.18. Satureja hortensis L. Essential Oil

*Satureja hortensis* L. belongs to the Lamiaceae family and is a well-known medicinal herb in Iran. Aerial parts of this plant are frequently used as a food additive and also as a traditional remedy to treat various disorders including cramps, muscle pain, nausea, indigestion, diarrhea and infectious diseases, based on the antispasmodic, antidiarrheal, antibacterial and antifungal properties of their constituents [140,141,142]. The essential oil (100 and 200 µL/Kg) from the seeds of *Satureja hortensis* in mice subjected to the acetic acid-induced writhing and formalin tests was tested [143]. A chemical analysis indicated the presence of γ-terpinene (50.5%) and thymol (32.7%) as the main constituents. In the acetic acid-induced writhing test, the essential oil significantly inhibited abdominal writhes. In the formalin test, the essential oil presented antinociceptive activity only in the late phase. Acetic acid-induced abdominal pain is not a specific model, but because of its similarity to the signs of human visceral disorders it has been extensively used for the screening of analgesic drugs [78,144]. In this test, many drugs including opioids, nonsteroidal anti-inflammatory drugs, antispasmodics, calcium channel blockers and antihistamines show analgesic activity [57,78]. Pain in the early phase is predominantly caused by the activation of C-fibers, while in the late phase, a combination of an inflammatory reaction in peripheral tissue and functional changes in the dorsal horn of the spinal cord are involved [65]. Finally, the authors suggested that the antinociceptive effect of the essential oil is dependent upon peripheral mechanisms.

### 3.19. Senecio rufinervis D.C. Essential Oil

*Senecio rufinervis* D.C. (Asteraceae) is a tall aromatic herb, leaves are shortly stalked, ovate, long pointed, sharply toothed; the lower surface is white and tomentose except for the nerves. The flowers are yellow and present as small rounded corymbs [145]. The plant grows in Uttarakhand, India at an altitude of 1800–3000 m and has no traditional or commercial use. *Senecio rufinervis* is an aromatic plant containing essential oil which is produced by many plants and confirms analgesic and antiinflammatory activities [146,147,148,149,150,151]. It was studied the antinociceptive effect of the essential oil from the dried leaves of *Senecio rufinervis* (25, 50 and 75 mg/Kg, i.p.) in mice subjected to the acetic acid-induced writhing and hot-plate tests [46]. A chemical analisys of the essential oil showed the presence of germacrene (40.19%) as the major constituent, followed by β-pinene (12.23%), β-caryophyllene (6.21%) and β-longipinene (4.15%). In the acetic acid-induced writhing test, the essential oil significantly and dose-dependently inhibited the acetic acid-induced abdominal twitches. Similarly, the essential oil significantly increased the latency of reaction time 15 and 30 min after the administration of drug. Therefore, Mishra *et al.* [46] demonstrated significant analgesic activity in both pain models. The acetic acid-induced writhing reaction in mice, described as a typical model for inflammatory pain, has long been used as a screening tool for the assessment of analgesic or anti-inflammatory properties of new agents [152]. The hot-plate method is considered to be selective for screening of the compound acting through the opioid receptor, but other centrally acting drugs, including sedatives and muscle relaxants, have also shown activity in this test [153]. From the above results the authors affirm that the oil possesses both peripheral and central analgesic effect. Yet, the peripheral analgesic effect produced by oil was more pronounced than the central analgesic effect.

### 3.20. Tetradenia riparia (Hochst.) Codd Essential Oil

*Tetradenia riparia* (Hochst.) Codd (Lamiaceae) is an herbaceous shrub that occurs throughout tropical Africa [154,155]. *T. riparia* possesses a variety of medicinal properties. In South Africa, *T. riparia* has traditionally been used in the treatment of cough, dropsy, diarrhea, fever, headaches, malaria and toothache [154]. In Brazil, *Tetradenia riparia* was introduced as an exotic ornamental plant and is grown in parks, home gardens and orchards in the state of São Paulo. In Brazil, it is popularly known as incenso, lavândula, limonete, pluma-de-névoa, or falsa mirra and is mainly used as an ornamental [155]. The antinociceptive effect of the essential oil obtained from the leaves of *Tetradenia riparia* (10 mg/Kg, p.o.), collected in different seasons, was investigated by Gazim *et al.* [47] in mice subjected to the acetic acid-induced writhing test. GC-MS analysis indicated that oxygenated sesquiterpenes were the dominant compounds, such as 14-hydroxy-9-epi-caryophyllene (18.27%–24.36%), *cis*-muurolol-5-en-4-α-ol (7.06%–13.78%), α-cadinol (5.36%–8.33%) and ledol (4.39%–8.74%). The content of the essential oil varied significantly by season. The oxygenated sesquiterpenes that varied most by season were 14-hydroxy-9-*epi*-caryophyllene (maximum 24.36% in spring, and absent in winter) and *cis*-muurolol-5-en-4-α-ol with a maximum of 13.78% in autumn and a minimum of 7.06% in winter. In the acetic acid-induced writhing test, the essential oil inhibited constrictions and this activity was not affected by seasonal variation. However, no additional information about the mechanism of action is given by the authors.

### 3.21. Teucrium stocksianum Essential Oil

The Lamiaceae family is rich in essential oils. The main components of the essential oil reported from the genus Teucrium are alpha pinene, linalool, carophyllene oxide, germacrene D, beta-carophyllene and delta-cadinene. *Teucrium stocksianum* is a species found in North Western Pakistan. It is a perennial aromatic herb of 10–30 cm height having grayish-white leaves and sessile flowers. It grows in the shades of the mountains. This plant is used in folk medicine for treating diarrhea, cough, jaundice and abdominal pain [156]. In the work developed by Shah *et al.* [48], a GC-MS analysis of the essential oil obtained from the aerial parts of *Teucrium stocksianum* revealed the presence of δ-cadinene (12.92%), α-pinene (10.3%), myrcene (8.64%), β-caryophyllene (8.23%), germacrene D (5.18%), limonene (2.36%), elemol (2.13%) and γ-cadinene (1.86%) as the main compounds. The antinociceptive activity of the essential oil (20, 40, 80 and 160 mg/Kg, i.p.) was assessed in mice subjected to the acetic acid induced writhing test. In this model of pain, the oil decreased the number of writhings. The acetic acid induced writhing protocol is most commonly used for evaluating antinociceptive activity of medicinal plants. In this model, prostaglandins, initially PGE2 and then PGF2α and free arachidonic acid are released from tissue phospholipids and, consequently, their levels in the peritoneal fluids increase due to intraperitoneal administration of the irritant, acetic acid. This results in localized inflammatory response and pain sensation due to increases in capillary permeability. Substances which counteract this phenomenon exert antinociceptive effects and reduce pain sensations [64]. Despite reporting these effects, more investigations are needed in order to elucidate the mechanism of action of the material.

### 3.22. Ugni myricoides (Kunth) O. Berg Essential Oil

The Myrtaceae family comprises a large number of plants, including at least 138 genera and approximately 3800 species [157,158]. Several experimental studies have demonstrated the biological activity of extracts, essential oils, or fractions obtained from different species of this family, such as *Psidium guajava* L., *Syzygium jambos* (L.) Alston, and *Plinia glomerata* (O. Berg) Amshoff [3,4,5,6]. *Ugni myricoides* (Kunth) O. Berg (syn. *Myrtus myricoides* Kunth), one of the four members of this genus, is found in Brazil, Southern Mexico, Central America, Colombia, Venezuela, Guyana, Ecuador and Peru [159]. The plant can be recognized by its small (1–1.5 cm long), opposite, coriaceous, dark green, aromatic, obovate to elliptic leaves and by its solitary white or pink flowers. The spherical fruits, of about 1 cm in diameter, are edible, fleshy and purple to black in color. Quintão *et al.* [49] studied the antinociceptive effect of essential oil from the leaves of *Ugni myricoides* (Kunth) O. Berg (5, 10, 12.5, 25 and 50 mg/kg, p.o.) in mice subjected to the following pain models: carrageenan- and CFA (complete Freund’s adjuvant)-induced mechanical hypernociception (evaluated by Von Frey hairs-induced hindpaw withdrawal response method), and used partial ligation of sciatic nerve tests. A GC-MS analysis of the essential oil indicated the presence of α-pinene (52.1%), 1,8-cineole (11.9%), α-humulene (4.6%), caryophyllene oxide + globulol (4.5%), humulene epoxide II (4.2%) and β-caryophyllene (2.9%) as the main components. In the first test, oral treatment with the essential oil was able to significantly inhibit the mechanical hypernociceptive response induced by carrageenan. This effect was observed for up to 48 h after treatment with the essential oil. Similar results were obtained with animals injected with CFA, in which *Ugni myricoides* (Kunth) O. Berg essential oil postponed the onset of hypernociceptive threshold for up to 24 h after CFA paw injection [49]. In addition, the hypernociceptive response evoked by CFA in the mouse paw was strikingly reduced by the pretreatment of animals with the pure monoterpene compound present as the major constituent in the oil, α-pinene (5–50 mg/Kg, p.o.), given 24 h before the injection of CFA. In the partial ligation of sciatic nerve tests, the essential oil was capable of diminishing the hipernociceptive response induced by this chronic constriction injury. This effect was observed for up to two days after the end of the treatment, and α-pinene administration was also capable of abolishing the hypernociceptive response in this model of pain. Quintão *et al.* [49] suggest that oral treatment with *Ugni myricoides* essential oil presents important effects in preventing and also reverting mechanical sensitization caused by inflammatory and neuropathic states. This conclusion is supported by results showing that the mechanical hypernociception induced by i.pl. injection of carrageenan or CFA in mice was strikingly reduced by both the essential oil and its major constituent. The injection of carrageenan into the hindpaws of mice induces a local inflammatory response, characterized by paw edema, neutrophil migration, and the release of several mediators such as cytokines, which precedes inflammatory hypernociception [160]. Additionally, CFA produces an inflammatory response that is associated with a striking modification in the activity of superficial (I and II), and deep (V and VI) laminal dorsal horn neurons receiving noxious inputs [161]. Also, chronic constriction nerve injury (such as partial ligation of the sciatic nerve) produces an inflammatory response that is associated with modification of the spinal cord neurons, culminating in altered neuronal excitability and conduction during evoked and spontaneous activity [162,163]. Quintão *et al.* [49] conclude that the essential oil obtained from the leaves of *Ugni myricoides* has relevant oral anti-hypernociceptive properties for persistent models of inflammatory and neuropathic pain in mice. However, the mechanism through which *Ugni myricoides* essential oil exerts its anti-hypernociceptive actions remains unclear and requires further investigation.

### 3.23. Valeriana wallichii DC Essential Oil

*Valeriana* (Valerianaceae) originated from the Latin word “valere” meaning “to be in good health”, as a source of important phytomedicines, has been used for curing nervous unrest, emotional troubles, epilepsy, insanity, snake-poisoning, eye-trouble and skin diseases [164]. *Valeriana wallichii* DC grows wild in the temperate Himalaya at an altitude of 1500–3000 m and is an ingredient of herbal medicines in Indian systems of medicine. Sah *et al.* [50] evaluated the antinociceptive effect of the essential oil isolated from the roots and rhizomes of *Valeriana wallichii* DC chemotype (patchouli alcohol) (20, 40 and 80 mg/Kg, p.o.) in mice subjected to the acetic acid induced writhing and tail-flick tests. GC-MS analysis of the essential oil revealed the presence of δ-guaiene (10%), seychellene (8%), acetoxyl patchouli alcohol (5%) and virdifloral (5%). Initially, the administration of the essential oil produced a significant inhibition of writhings. The effect was potentiated in the presence of aspirin (5 mg/Kg, i.p.). The writhing test induced by acetic acid in mice is described as a typical model of study of inflammatory pain, being used as a screening for evaluation of analgesics or anti-inflammatory drugs [131]. The local irritation provoked by intraperitoneal injection of acetic acid triggers liberation of a variety of mediators such bradykinin, substance P and prostaglandins, (especially PGI2), as well as certain cytokines such as IL-1β, TNF-α and IL-8 [132]. Such mediators activate chemosensitive nociceptors that contribute to the development of this type of inflammatory pain, which is known to be sensitive to non-steroidal anti-inflammatory drugs (NSAIDs). However, the essential oil failed to prolong the latency time in the tail-flick model. This pain model is reported to be a useful test to discriminate analgesic agents acting primarily at the spinal medulla level and at the higher central nervous system levels, from those acting by peripheral mechanisms, (positive results indicating central action [3]. Based on the results, Sah *et al.* [50] suggest that *Valeriana wallichii* DC essential oil possesses peripheral analgesic action and the effect is comparable to aspirin.

### 3.24. Xylopia laevigata (Mart.) R.E.Fries Essential Oil

The Annonaceae is a large family of tropical and subtropical trees and shrubs, comprising about 135 genera and more than 2500 species [165,166]. This family is known for its edible fruits and the medicinal properties of many of its species [167]. In the Brazilian Northeast, *Xylopia laevigata* (Mart.) R.E. Fries (Annonaceae) is commonly called “meiú” or “pindaíba”, a plant (leaves and flowers) used popularly to treat painful disorders, heart disease and inflammatory conditions [51]. Queiroz *et al.* [51] evaluated the antinociceptive effect of *Xylopia laevigata* essential oil in mice subjected to the acetic acid-induced writhings and formalin tests. Chemical analysis indicated the presence of the following sesquiterpenes: γ-muurolene (17.78%), δ-cadinene (12.23%), bicyclogermacrene (7.77%), α-copaene (7.17%), germacrene D (6.54%), (*E*)-caryophyllene (5.87%), γ-cadinene (4.72%), aromadendrene (4.66%) and γ-amorphene (4.39%). When tested, the essential oil significantly inhibited the acetic acid-induced writhings, and the two phases of formalin. It is important to stress that the antinociceptive effect of the oil was not reversed by naloxone (1.5 mg/Kg, i.p.) in the formalin test. The acetic acid-induced abdominal constriction test is a standard, simple and sensitive model for measuring analgesia induced by both opioids and peripherally acting analgesics [168]. According to Le Bars *et al.* [3], in the acetic acid test, pain is elicited by the injection of an irritant, such as acetic acid into the peritoneal cavity, which produces episodes of characteristic stretching (writhing) movements; those behavioral changes are probably in relation to the inhibition in the peritoneal fluid levels of prostaglandin and cytokines. This information supports the authors’ conclusion that the essential oil may also participate in the inhibition of prostaglandin synthesis, since nociceptive mechanisms involve the processing or release of arachidonic acid metabolites via COX and prostaglandin biosynthesis [169]. In addition, the formalin test is sensitive to various classes of analgesic drugs [24]; and is characterized by the first phase (neurogenic), which is evoked by direct formalin stimulation of the sensorial C-fibers followed by substance P release [60], and the second phase (inflammatory) mainly due to a subsequent inflammatory reaction in the peripheral tissue mediated by the release of various inflammatory mediators associated with the increased level of prostaglandin, induction of COX and release of nitric oxide (NO) [3]. It is important to note that the essential oil reduced the production of nitrite, showing it to have a potential role as a NO scavenging agent, and since NO plays an important role in various types of inflammatory processes, it is thus possible that the reduction of NO is involved in a potential antinociceptive action of the *Xylopia laevigata* essential oil [51].

### 3.25. Vanillosmopsis arborea Baker Essential Oil

*Vanillosmopsis arborea* Baker is native to the Araripe National Forest, in the Northeast of Brazil in the state of Ceará. There are few studies concerning the traditional use of this plant. However, biological and pharmacological studies have shown that its essential oil presents antimicrobial, antiinflammatory and gastroprotective activities [170]. The topical antinociceptive effect of *Vanillosmopsis arborea* Baker essential oil (25, 50, 100 and 200 mg/Kg, p.o. or topical) was studied by Leite *et al.* [52] in mice subjected to formalin and eye wiping (corneal nociception) tests. The composition (*w*/*w*) of *Vanillosmopsis arborea* Baker essential oil revealed the presence of α-bisabolol (70%). Other identified compounds were α-cadinol (8.4%), elemicin (6.21%), β-bisabolene (4.46%), δ-guaiene (2.31%), β-cubebene (1.76%) and estragole (1.08%). In the formalin test, pretreatment with the essential oil (oral and topical) caused significant reductions of both first phase (neurogenic) and second phase (inflammatory) nociception responses. Such effect may be related, at least in part, to release of leukotrienes, which decrease the production of inflammatory eicosanoids and influence the production of arachidonic acid metabolites [171]. This antinociceptive effect may also be related to the high α-bisabolol content in the essential oil, since α-bisabolol possesses visceral antinociceptive activity [172], and is able to reduce neuronal excitability in a concentration-dependent manner [107]. The topically administered essential oil decreased the number of eye wipes induced through local application of 5 M NaCl solution on the corneal surface. Oral treatment with the oil also reduced the number of eye wipes. In addition, the antinociceptive effect induced by the essential oil was significantly inhibited by ondansetron (0.5 mg/Kg, i.p.), PCPA (a tryptophan hydroxylase inhibitor—100 mg/Kg, i.p.), prazosin (0.15 mg/Kg, i.p.), atropine (0.1 mg/Kg, i.p.) and capsazepine (5 mg/Kg, i.p.). On the other hand, the administration of glibenclamide (2 mg/Kg, i.p.), naloxone (2 mg/Kg, i.p.), ruthenium red (5 mg/Kg, s.c.), yohimbine (2 mg/Kg, i.p.), L-NAME (2 mg/Kg, i.p.) or theophylline (5 mg/Kg, i.p.) did not prevent the essential oil-induced antinociception. The cornea is used for nociception studies on the trigeminal system [173], since corneal nociceptive receptors have large representation in the trigeminal ganglion through the ophthalmic branch of the trigeminal nerve [174]. Thin myelinated fibres [175] as well as unmyelinated fibers in the cornea respond to chemical, mechanical and thermal noxious stimuli [176]. The application of hypertonic saline to the tongue and cornea transiently activates nociceptive neurons with wide dynamic range properties in the trigeminal subnucleus caudalis [177]. Moreover, infusion of hypertonic saline into the masseter muscle produces hind paw shaking and activates c-Fos positive neurons in the ipsilateral trigeminal subnucleus caudalis [178]. Taken together, the results indicate that the essential oil of *Vanillosmopsis arborea* Baker exerts antinociceptive activity by peripheral and central mechanisms, possibly mediated by 5-HT, α1, muscarinic and TRPV1 receptors.

### 3.26. Zingiber oficinalle and Zingiber zerumbet Essential Oils

The Zingiberaceae family is among the most prolific plants in tropical rainforests. Ginger, the rhizome of *Zingiber officinale* Roscoe, is one of the most widely used spices and a traditional remedies in Indian, Chinese and Oriental medicine against pain, inflammation and gastrointestinal disorders. Ginger oil is produced from fresh rhizomes of *Zingiber officinale*. It possesses the aroma and flavor of the spice but lacks the pungency. The essential oil of ginger has been found to possess antibacterial, antiviral and antifungal properties [179,180]. *Zingiber zerumbet* (L.) Smith, locally known in Malaysia as “lempoyang” is one of the commonly used wild ginger species in Malay traditional medicine. The concoction of *Zingiber zerumbet* rhizomes is normally drunk to treat indigestion, stomach ache, fever, and worm infestation. The young stems, rhizomes and inflorescence are also used as a poultice for topical applications to treat muscle sprain and as a curative for swelling sores. The juice extracted from the rhizomes or the cooked rhizomes are usually taken by women post-partum or post-surgical patients to improve appetite, enhance recovery or healing as well as to alleviate pain [181,182]. Jeena *et al.* [53] studied the antinociceptive effect of the essential oil of ginger (100, 500 and 1000 mg/Kg, i.p.) in mice subjected to the acetic acid-induced writhing test. The principal constituent of ginger oil was found to be zingiberene (31.08%), a sesquiterpene hydrocarbon, followed by arcurcumene (15.4%) and α-sesquiphellandrene (14.02%). Other compounds include bisabolene (13.80%) and sabinene (8.27%). Ginger oil showed marked and significant reduction in the number of writhings induced by acetic acid. The analgesic activity at all tested doses indicated a dose dependent relationship. Jeena *et al.* [53] affirm that acetic acid induces pain in the peritoneal cavity by enhancing levels of endogenous substances like: PGE2 and PGF2 [63]. This indicates that acetic acid acts indirectly in the stimulation of nociceptive neurons by releasing endogenous mediators and suggests that ginger oil has strong antinociceptive activity. Its mode of action might involve inhibition of arachidonic acid synthesis, a metabolite mediated by COX inhibition. Sulaiman *et al.* [54] investigated the antinociceptive effect of essential oil from the rhizome of *Zingiber zerumbet* (30, 100 and 300 mg/Kg, i.p. and p.o.) in mice subjected to the following pain models: acetic acid-induced abdominal writhing, formalin and hot-plate tests. GC/MS analyses indicated the presence of zerumbone (36.12%) was the most abundant constituent among the sesquiterpenes, followed by humulene (10.03%), humulene oxide I (4.08%), humulene oxide II (2.14%), caryophyllene oxide II (1.66%) and caryophyllene oxide I (1.43%). Among the monoterpenes we found: camphene (14.29%), borneol (4.78%), camphor (4.18%), eucalyptol (3.85%), α-pinene (3.71%), γ-terpinene (2.00%), β-phellandrene (1.63%), 1-terpen-4-ol (1.44%), β-myrcene (1.22%) and linalool (1.06%). Intraperitoneal administration of the essential oil caused dose-dependent inhibition of the writhing response induced by acetic acid. The oral administration caused a partial but significant inhibition of the acetic acid-induced pain. This method is very sensitive and able to detect antinociceptive effects of compounds and dose levels that may appear inactive in other methods like the tail-flick test [183]. It has been suggested that acetic acid acts indirectly by releasing endogenous mediators, such as PGE2 and PGF2α as well as increasing lipoxygenase production in the peritoneum that stimulate the nociceptive neurons sensitive to nonsteroidal anti-inflammatory drugs [184]. Therefore, the results of the acetic acid-induced abdominal constriction test strongly suggest that the mechanism of action of the oil may be mediated by lipoxygenases and/or cyclooxygenases’ activity inhibition. In the formalin test, intraperitoneal pretreatment with different doses of *Zingiber zerumbet* essential oil had significant and dose-dependent effects on the duration of licking activity in both early and late phases of the test. Such effect was reversed significantly by naloxone (5 mg/Kg, i.p.). It is well known that centrally acting drugs such as narcotics inhibit both nociception phases equally, while peripherally acting drugs such as acetylsalicylic acid, which block prostaglandin synthesis, only inhibit the second phase [60]. Taken together, Sulaiman *et al.* [54] affirm that the antinociceptive effects of the essential oil in the writhing test and in both phases of the formalin test strongly suggested that they contained active analgesic principles acting both centrally and peripherally, it was also implied that the extract possessed not only antinociceptive but also antiinflammatory activity. This finding is supported, at least in part, by the results of the hot plate test. In this pain model, the intraperitoneal administration of the oil increased the latency time to the nociceptive response in the hot plate test significantly. This effect began early, 30 min after intraperitoneal administration of the essential oil, and persisted until the fifth hour. The antinociceptive effect was also reversed by naloxone. In a later study, Khalid *et al.* [55] suggested a mechanism of antinociceptive action for *Zingiber zerumbet* essential oil (50, 100, 200 and 300 mg/Kg, i.p. and p.o.). Acetic acid-induced abdominal constriction, capsaicin-, glutamate- and phorbol 12-myristate 13-acetate-induced paw licking tests in mice were employed in the study. The essential oil exhibited significant dose-dependent inhibition on abdominal writhing when administered intraperitoneally. Similar dose dependent inhibition was also observed in mice administered orally. Likewise, intraperitoneal administration of *Zingiber zerumbet* essential oil at similar doses produced significant dose dependent inhibition of neurogenic pain induced by intraplantar injection of capsaicine (1.6 µg/paw). It is believed that capsaicin directly activates a non-selective ionotropic channel in primary sensory neurons, the capsaicin receptor, also known as the transient receptor potential vanilloid 1 (TRPV1) [185]. Therefore, this finding indicates that the effect of the essential oil may involve, at least in part, TRPV1 receptor inhibition. Similarly, the essential oil also inhibited pain induced by intraplantar injection of glutamate (10 µM/paw). It was reported that this nociceptive response caused by glutamate involves peripheral, spinal and supraspinal sites of action with glutamate receptors (AMPA, kainate and NMDA receptors), which play an important role in modulating the nociceptive response [186]. A similar result was observed with intraplantar administration of phorbol 12-myristate 13-acetate (a PKC activator at 1.6 µg/paw). PKC activation is an essential step for the nociceptive effects of numerous stimuli, including those that are caused by inflammatory mediators. PKC phosphorylates many cellular components, including membrane bound receptors, ion channels and enzymes, which are known to regulate the excitation of nociceptors [187]. Peripheral introduction of PMA produces nociception, thermal hyperalgesia and mechanical allodynia in mice and rats. PMA, acting on PKC can directly stimulate TRPV1 channels leading to the propagation of nociceptive impulses [188]. This finding can be linked to earlier findings discussed above. It was also demonstrated that pretreatment with l-arginine (100 mg/Kg, i.p.) significantly reversed the antinociceptive activity induced by the oil. Previous studies have reported that NOS inhibitors reduced nociception caused by acetic acid [189]. NO is a diffusible gas that permeates cell membranes and is not stored in vesicles. NMDA receptor activation increases intracellular calcium, which in turn stimulates NOS to catalyze the substrate l-arginine to NO and to l-citrulline [190]. NO seems to be involved in all three levels of the pain pathway, which are the peripheral, spinal cord dorsal horn and the cerebral cortex where perception of pain is processed [191]. In addition, pre-treatment with methylene blue (20 mg/Kg, i.p.) significantly enhanced antinociception produced by the essential oil. It has been suggested that methylene blue promotes antinociception by sequentially inhibiting peripheral NOS and guanylyl cyclase, resulting in NO interference [192]. The activation or deactivation of noci-responsive neurons is dependent on the availability of cGMP. Intracellular cGMP concentrations are regulated by the action of guanylyl cyclase and also by the rate of degradation by cGMP-specific phosphodiesterases [193]. Therefore, cGMP is very important for the functioning of nociceptors. Nitric oxide activates guanylyl cyclase, which in turn catalyses the formation of cGMP from guanosine triphosphate, whereas cyclic GMP-specific phosphodiesterase catalyzes the hydrolysis of cGMP to GMP, thus consequently ending the signal transduction [194]. Finally, the administration of glibenclamide (10 mg/Kg, i.p.), an ATP-sensitive K^+^ channel antagonist, significantly reversed antinociceptive activity induced by the essential oil. This finding suggests that the oil exerted its antinociceptive activity through the opening of ATP sensitive K^+^ channel that allows the efflux of K^+^ ions, thus leading to membrane repolarization and/or a hyperpolarization state which reduces the membrane excitability [195]. Taken together, the results indicate that the antinociceptive action of *Zingiber zerumbet* essential oil involves, at least in part, the activation of the l-arginine/NO/cGMP/ATP-sensitive K^+^ channel pathway, apart from its ability to interact and inactivate the TRPV1 receptor, inactivation of protein kinase C, and also inhibition of the glutamatergic system.

## 4. Conclusions and Perspectives

In conclusion, many aromatic plant species have essential oils that present antinociceptive activity. Testing suggests that the antinociceptive effect of essential oils is due to their major constituents and that synergism between such chemical constituents does occur. Antinociceptive activity of essential oils involves peripheral (antiinflammatory), and/or central mechanisms. In many cases, the central mechanism involves the opioid system. However, in some studies, the cannabinoid, glutamatergic or nitrergic pathways are involved. Data from this review show the potential of essential oils like low cost analgesic drugs for new treatments for pain. However, there are few reports of toxicity to confirm therapeutic safety and to carry out clinical tests.

## Abbreviations

5-HT: 5-hydroxytryptamine
AMPA: α-amino-3-hydroxy-5-methyl-4-isoxazolepropionic acid
CB1/CB2: cannabinoid receptor type 1 and 2
CFA: complete Freund’s adjuvant
cGMP: cyclic guanosine monophosphate
COX: cyclooxygenase
GABA: gamma-Aminobutyric acid
GC/MS: gas chromatography/mass spectrometry
HPLC: high performance liquid chromatography
IL-1β: interleukin 1β
IL-8: interleukin 8
L-NAME: *N*-nitro-l-arginine methyl Ester
NMDA: *N*-methyl d-aspartate
NO: nitric oxide
NOS: nitric oxide synthase
NSAIDs: non-steroidal aintiinflammatory drugs
PCPA: *p*-chlorophenylalanine
PGE2: prostaglandin E2
PGF2α: Prostaglandin F2α
PGI2: prostacyclin
PGs: prostaglandins
PKC: protein kinase C
PMA: phorbol 12-myrstrato 13-aspartate
TNFα: tumor necrosis factor α
TRPV1: transient receptor potential vanilloid 1
